# Complete mitochondrial genome and the phylogenetic position of the Burmese narrow-headed softshell turtle *Chitra vandijki* (Testudines: Trionychidae)

**DOI:** 10.1080/23802359.2021.1903350

**Published:** 2021-03-26

**Authors:** Chen Chen, Hong Xiaoyou, Li Wei, Yu Lingyun, Chen Haigang, Zhu Xinping

**Affiliations:** Key Laboratory of Tropical and Subtropical Fishery Resources Application and Cultivation, Ministry of Agriculture and Rural Affairs, Pearl River Fisheries Research Institute, Chinese Academy of Fishery Sciences, Guangzhou, China

**Keywords:** Mitochondrial genome, conservation, Chitra *vandijki*, softshell turtle

## Abstract

Narrow-headed softshell turtles constitute a group of critically endangered freshwater turtles that belong to the family Trionychidae. Here, we determine the complete mitogenome of the Burmese narrow-headed softshell turtle *Chitra vandijki*. The length of the mitochondrial genome was 16,614 bp, composed of 13 protein-coding genes, 22 tRNA genes, two rRNA genes, and twelve noncoding regions. The phylogenetic analysis strongly indicated that *C. vandijki* is closely related to *C. indica*. The mitochondrial genome will contribute to the genetic research and conservation of *C. vandijki* in the future.

The Burmese narrow-headed softshell turtle *Chitra vandijki* McCord and Pritchard (2002), named Peter-Paul van Dijk, is the largest *Chitra* species (McCord and Pritchard 2002). *C. vandijki* experienced allopatric speciation around the Ayeyarwaddy River basin of Myanmar throughout evolution. This gives the species unique cephalic and neck patterns compared to the other three congeners (McCord and Pritchard 2002). In recent years, due to overexploitation and expansion of the international turtle trade, declining population has seriously threatened the persistence of *C. vandijki*. The situation has highlighted the need for protective actions. The IUCN/SSC Tortoise and Freshwater Turtle Specialist Group (TFTSG) categorized the species as critically endangered in 2011.

Mitogenomic information is considered a valuable molecular tool in species identification and phylogenetic conservation research. For this reason, we determined the complete mitochondrial genome of *C. vandijki* and analyzed it by comparing it with that of other Trionychidae turtles to confirm the phylogenetic relationship between them.

An umbilical cord tissue sample was carefully taken from a hatchling turtle bred from two captive *C. vandijki* individuals at Xishuangbanna, Yunnan, China (22°0′N, 100°47′E) in October 2019. The tissue sample was stored in 95% ethanol solution and deposited in our laboratory specimen bank (Dr. Chen, chenchen3729@outlook.com) under voucher number PRFRI_CVAND_20191001.

Total genomic DNA was extracted from the ethanol-preserved specimen using a MicroElute Genomic DNA kit (Omega, USA), and a 350-bp DNA library was constructed. Sequencing was performed on an Illumina HiSeq Xten platform (Illumina, USA), and a dataset of 38,530,879 raw reads generated. After trimming adapters and quality filtering, the clean data were assembled using SPAdes software (Bankevich et al. 2012). The mitochondrial genome was annotated through the use of MITOS (http://mitos.bioinf.uni-leipzig.de/ index.py) (Bernt et al. 2013), NCBI ORF finder, and tRNA-scan SE (http://lowelab.ucsc.edu/tRNAscan-SE/) (Lowe and Eddy 1997). After manual examination to ensure its correct assembly, the complete annotated *C. vandijki* mitogenome was submitted to GenBank under accession number MT683848.

All 13 mitochondrial protein-coding genes (PCGs) in 27 turtles were chosen (Supplementary Table S2) to investigate the phylogenetic position of *C. vandijki*, with *Mauremys reevesii* (NC_006082) and *Mauremys mutica* (NC_009330) as the outgroups. Each of the 13 PCG sequences was individually aligned with MAFFT (Katoh et al. 2005) by default settings, and ambiguous characters were removed via GBlocks (Castresana 2000). Then, 13 PCG sequences were concatenated in BioEdit (Hall 1999). Next, the optimal partitioning scheme and substitution model were determined by using PartitionFinder (Lanfear et al. 2016). Phylogenetic trees were reconstructed by IQ-TREE (Nguyen et al. 2015) via the maximum likelihood (ML) method in partition mode (Supplementary Table S3). Node support was calculated with 5000 bootstrap replications.

The total mitochondrial genome of *C. vandijki* was 16,614 bp in length with 40.41% CG content and consisted of 13 PCGs, two rRNA genes, 22 tRNA genes, and 12 noncoding regions. Twelve *C. vandijki* PCGs initiated with ATG start codons, and one initiated with GTG (*cox1*). Nine *C. vandijki* PCG stop codons were complete, including seven terminated by TAA, one ended with AGA (*nad6*), and one ended with AGG (*cox1*). The remaining four *vandijki* PCG stop codons were incomplete, ending with T– (*nad2*, *cox3*, *nad3*, and *nad4*). Similar to other Trionychidae turtles, the *C. vandijki* 12S rRNA genes (970 bp) and 16S rRNA genes (1595 bp) were distributed between tRNA^Phe(UUC)^ and tRNA^Val(GUA)^, and between tRNA^Val(GUA)^ and tRNA^Leu (UUA)^, respectively. The two long noncoding regions were O_L_ (29 bp) and the control region (1115 bp), whereas the lengths of the other ten regions were small, ranging from 1 to 11 bp with a total length of 40 bp (Supplementary Table S1).

The ML phylogenetic tree best supported that *C. vandijki* belonged to Trionychinae and was closely related to *Chitra indica* with 100% bootstrap support (Figure 1). This result is consistent with that of Engstrom et al. (2002) and McCord and Pritchard (2002), which was based on the likelihood and parsimony analysis of the ND4 gene. Our mitochondrial genome sequence data provide a new source of useful information for the genetic research and conservation of *C. vandijki*.

**Figure 1.**
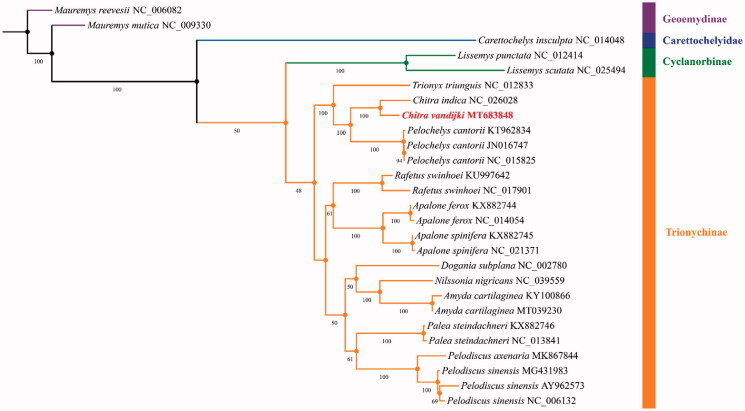
Maximum-likelihood (ML) tree showing the phylogenetic relationship of 27 turtles based on the concatenated nucleotide sequences of all 13 mitochondrial protein-coding genes (PCGs), with the Mauremys reevesii (NC_006082) and the Mauremys mutica (NC_009330) as outgroups. GenBank accession numbers of each sequence were next to the species name, and the numbers at each node represented the bootstrap support values (%) based on 5000 replicates. The Chitra vandijki was in bold red font.

## Data Availability

The genome sequence data that support the findings of this study are openly available in GenBank of NCBI at (https://www.ncbi.nlm.nih.gov/) under the accession no. MT683848. The associated BioProject, SRA, and Bio-Sample numbers are PRJNA706680, SUB9182104, and SAMN18140118 respectively.
